# A Mediterranean diet plan in lactating women with obesity reduces maternal energy intake and modulates human milk composition – a feasibility study

**DOI:** 10.3389/fnut.2024.1303822

**Published:** 2024-03-13

**Authors:** Clark R. Sims, Jessica L. Saben, Audrey Martinez, Sarah R. Sobik, Meghan R. Crimmins, Jessica E. Bulmanski, Donald Turner, Annalee Furst, Lisa T. Jansen, Lars Bode, Aline Andres

**Affiliations:** ^1^Department of Pediatrics, University of Arkansas for Medical Sciences, Little Rock, AR, United States; ^2^Arkansas Children’s Nutrition Center, Little Rock, AR, United States; ^3^Department of Surgery, University of Colorado Anschutz Medical Campus, Aurora, CO, United States; ^4^Colorado Center for Transplantation Care, Research, and Education, University of Colorado Anschutz Medical Campus, Aurora, CO, United States; ^5^Department of Physiology and Cell Biology, University of Arkansas for Medical Sciences, Little Rock, AR, United States; ^6^College of Medicine Office of Research, University of Arkansas for Medical Sciences, Little Rock, AR, United States; ^7^Department of Pediatrics, School of Medicine, Larsson- Rosenquist Foundation Mother-Milk-Infant Center of Research Excellence, University of California, San Diego, La Jolla, CA, United States; ^8^Department of Dietetics and Nutrition and Department of Pediatrics, University of Arkansas for Medical Sciences, Little Rock, AR, United States

**Keywords:** human milk, obesity, Mediterranean meal plan, human milk oligosaccharides, infant growth, healthy eating index, maternal diet

## Abstract

**Introduction:**

Maternal obesity is associated with increased concentrations of human milk (HM) obesogenic hormones, pro-inflammatory cytokines, and oligosaccharides (HMOs) that have been associated with infant growth and adiposity. The objective of this pilot study was to determine if adherence to a Mediterranean meal plan during lactation modulates macronutrients and bioactive molecules in human milk from mothers with obesity.

**Methods:**

Sixteen healthy, exclusively breastfeeding women with obesity (body mass index ≥30 kg/m^2^) enrolled between 4 and 5 months postpartum. The women followed a 4-week Mediterranean meal plan which was provided at no cost. Maternal and infant anthropometrics, HM composition, and infant intakes were measured at enrollment and at weeks 2 and 4 of the intervention. Thirteen mother-infant dyads completed the study. Additionally, participants from an adjacent, observational cohort who had obesity and who collected milk at 5 and 6 months postpartum were compared to this cohort.

**Results:**

Participants’ healthy eating index scores improved (+27 units, *p* < 0.001), fat mass index decreased (−4.7%, *p* < 0.001), and daily energy and fat intake were lower (−423.5 kcal/day, p < 0.001 and-32.7 g/day, *p* < 0.001, respectively) following the intervention. While HM macronutrient concentrations did not change, HM leptin, total human milk oligosaccharides (HMOs), HMO-bound fucose, Lacto-N-fucopentaose (LNFP)-II, LNFP-III, and difucosyllacto-N-tetrose (DFLNT) concentrations were lower following the intervention. Infant intakes of leptin, tumor necrosis factor (TNF)-α, total HMOs, HMO-bound fucose, LNFP-III and DFLNT were lower following the intervention. Specific components of the maternal diet (protein and fat) and specific measures of maternal diet quality (protein, dairy, greens and beans, fruit and vegetables) were associated with infant intakes and growth.

**Discussion:**

Adherence to a Mediterranean meal plan increases dietary quality while reducing total fat and caloric intake. In effect, body composition in women with obesity improved, HM composition and infants’ intakes were modulated. These findings provide, for the first time, evidence-based data that enhancing maternal dietary quality during lactation may promote both maternal and child health. Longer intervention studies examining the impact of maternal diet quality on HM composition, infant growth, and infant development are warranted.

## 1 Introduction

In the United States, more than 50% of women enter pregnancy with either overweight or obesity (1). Pre-gravid obesity has been associated with changes in the macronutrient (2) and bioactive composition of human milk (HM) (2–4), which may impact infant health (5). HM from women with obesity has higher energy, fat, and protein content compared to milk from mothers with normal weight throughout lactation (2, 6, 7) and obesity-associated elevations in HM hormones (8, 9), pro-inflammatory cytokines (10, 11) and HM oligosaccharides (HMOs) (4), are positively associated with infant growth and adiposity (2, 4, 9). Therefore, obesity-associated alterations in HM composition may play a role in early-life nutritional programing of infant adiposity.

Dietary interventions during the postnatal period may provide a window to temper the effects of obesity on HM composition. It has been shown in observational studies that a higher Mediterranean diet score is associated with lower HM saturated fatty acid concentrations and with increased monounsaturated fatty acids and total antioxidant capacity (12, 13). However, very few dietary intervention studies have been conducted in breastfeeding women that have also analyzed components of HM. A crossover study, employing four different dietary paradigms (galactose vs. glucose and high carbohydrate vs. high fat) during lactation, showed an association between dietary energy source and HMO concentrations (14). Another study, aimed at decreasing maternal energy, fat, and sugar intake over 2 weeks postpartum, found that HM insulin, leptin and adiponectin were reduced by 10–25% following the dietary intervention (15). Together these studies suggest that dietary interventions can modulate HM composition. As such, the Mediterranean diet has shown efficacy in decreasing body mass index (BMI) (16, 17), circulating obesogenic hormones (17), adipokines (18), and systemic inflammation (16, 19) in non-pregnant/non-lactating women with obesity. However, it is yet unknown whether similar results can be attained in lactating women or if these changes may affect human milk content.

In this within-subject pilot intervention trial, we aimed to determine if adherence to a Mediterranean meal plan during lactation could modulate the macronutrient and bioactive (hormone, HMO, and cytokine) content of HM from women with obesity.

## 2 Materials and methods

### 2.1 Participants and study design

The within-subject intervention study took place at the Arkansas Children’s Nutrition Center in Little Rock, Arkansas between April 2019 and February 2020. Healthy women with obesity who were exclusively breastfeeding were recruited from the surrounding community. Of the 90 participants screened, 28 were eligible and of those, 16 enrolled between 4 and 5 months postpartum (Supplementary Figure S1). Three participants did not complete all study visits (19%), resulting in 13 participants for the current analysis. Inclusion criteria were: BMI = 30–50 kg/m^2^, ≥ 18 years of age, singleton pregnancy, intent to continue breastfeeding exclusively until at least 6 months postpartum, and child being able to be fed expressed milk from a bottle. Exclusion criteria included: pre-existing conditions (e.g., diabetes, hypertension, heart disease); use of recreational drugs, tobacco, or alcohol; food allergies, intolerances or preferences incompatible with meal plan; and the use of medications or supplements that are contraindicated for lactating mothers. Maternal age, race and ethnicity, and infant sex were self-reported. Assessments took place at enrollment (pre), 2 weeks and 4 weeks following the start of the dietary intervention (Wk2 and Wk4, respectively). To examine the impact of time on milk composition and infant intakes, participants from an adjacent, observational cohort from the same study center (2, 4, 20) were matched to the participants of the within-subject intervention study based on maternal BMI and HM sample availability at postpartum months 5 and 6. From the adjacent, observational study, there were only 10 participants that had a BMI above 30 and collected milk samples at both 5 and 6 months postpartum, therefore, all 10 were used to compare with the participants from this within-subject study. To learn about the observational study sample used, please refer to our group’s previous publications on this cohort (2, 4, 20).

### 2.2 Ethics statement

Written, informed consent was obtained from all participants prior to study procedures. All study procedures were approved by the Institutional Review Board of the University of Arkansas for Medical Sciences (Protocol #: 228407). This trial was registered at clinicaltrials.gov (NCT03744429).

### 2.3 3-day food records

Habitual maternal dietary intake was assessed prior to the initiation of the dietary intervention using 3-day food records (two weekdays, one weekend day) and analyzed with the Nutrition Data System for Research (Nutrition Coordinating Center, University of Minnesota, MN) software by trained interviewers. Participants recorded all food, beverages, supplements, and medications that they consumed during the 3 day period.

### 2.4 Dietary intervention

Participants met with a registered dietitian at the initial study visit to receive education about the dietary intervention based on the Mediterranean diet (21) and weekly thereafter to monitor adherence to the meal plan. Motivational interviewing, active listening, and goal setting techniques were used to help participants comply with the intervention. The goals of the counseling sessions were to identify and resolve barriers to adherence as well as provide encouragement and support. The initial session educated on the study intervention and tracking dietary intake while subsequent sessions reviewed compliance to problem solve challenges and celebrate successes. The macronutrient distribution (20–35% of calories from fat, 45–65% carbohydrates, 10–35% protein) and provided caloric intake met the Dietary Guidelines for Americans recommendations (22). All lunches and dinners (2/day, in the form of fresh packaged meals) were provided weekly to the participants throughout the 4 weeks by Trifecta Nutrition (Sacramento, California). Breakfast (breakfast sandwiches and oatmeal, 1/day) and snacks (walnuts, granola bars, Greek yogurt, and fruits, 2/day) were provided by the research team. Participants were also provided with extra virgin olive oil to add to their meals and were instructed to buy 1% low fat milk to drink or combine with fruits as a smoothie. Participants recorded all food, beverages, supplements, and medications that they consumed and where they made substitutions in the meal plan for the entirety of the trial. Dietary intake was analyzed using the Nutrition Data System for Research. Healthy Eating Index (HEI) and Mediterranean Diet scores were derived from published guidelines (23–25). The overall intervention dietary composition is summarized in Supplementary Table S1 and an example of a week’s menu is shown in Supplementary Table S2. Intervention compliance was calculated as the participants HEI score of consumed meals divided by the HEI score of the prescribed meals multiplied by 100.

### 2.5 Anthropometrics and body composition

Maternal and infant anthropometrics and maternal body composition were measured at each visit. Maternal weight and height and infant weight and length were measured as previously described (2). Weight-for-length, weight-for-age and length-for-age z-scores were calculated based on the World Health Organization Child Growth Standards (26, 27). Maternal BMI was calculated as kg/m^2^. Maternal fat mass (FM) and fat free mass (FFM) were measured using air displacement plethysmography (Cosmed BodPod®, Concord, CA). Infant fat mass and lean mass were measured using quantitative nuclear magnetic resonance (EchoMRI-AH, Echo Medical Systems, Houston, TX). FM and FFM index (FMI and FFMI, respectively) were calculated as FM (kg)/m^2^ and FFM (kg)/m^2^.

### 2.6 Plasma analysis

Maternal blood was collected following an overnight fast at the pre-intervention and Wk4 visits only. Plasma was processed and stored at −80°C. Leptin, insulin, C-reactive protein (CRP), interleukin (IL)-6, IL-8, and TNF-α concentrations were measured using high-performance electrochemiluminescence immunoassays (Meso Scale Diagnostics, Rockville, MD). Cholesterol, high-density lipoprotein (HDL), and low-density lipoprotein (LDL) concentrations were measured using a clinical analyzer (Randox Laboratories, Kearneysville, WV).

### 2.7 24-h human milk collection

Participants collected HM over 24-h, prior to each visit. Mothers were given the option to either feed their infant the expressed milk from a bottle or to feed baby from one breast and pump the other breast during the 24-h collection period. If only one breast was pumped, mothers were instructed to alternate the nursed breast and the pumped breast at each feed and record accordingly. At each feed, the mothers were asked to gently invert the expressed HM and aliquot 4 mL of HM into the provided polypropylene tubes. HM was stored at 4°C until the full 24-h collection was complete. Afterwards, the 24-h samples were pooled and stored intact at-80°C.

### 2.8 Human milk composition and infant intakes

Macronutrients (fat, protein, and carbohydrates) were measured in milk from all visits using a Miris HM Analyzer (Miris, Uppsala, Sweden) according to manufacturer’s instructions, from which caloric content was derived. Leptin, insulin, CRP, IL-6, IL-8, and TNF-α concentrations were measured in milk from all visits using high-performance electrochemiluminescence immunoassays (Meso Scale Diagnostics, Rockville, MD). Concentrations of HMOs (nmol/mL) were measured in milk from the pre-intervention and Wk4 visits only.by high-performance liquid chromatography on an amide-80 column (2 μm particle size, 2 mm ID, 15 cm length) with fluorescent detection, as previously described (14). The absolute quantification of the 19 most abundant HMOs (4) was determined using the non-HMO oligosaccharide raffinose as an internal standard added to all milk samples at the beginning of analysis. Infant intakes were estimated using test weighing, which is considered a useful and precise method for assessing milk intake (28–30), at each visit to obtain a single-feed milk intake volume multiplied by regular, daily feeding frequency as reported by the mothers.

### 2.9 Statistical analyses

Demographic data was summarized using mean and standard deviation for continuous variables and counts (percentages) for categorical variables. Comparisons were made using linear mixed-effect models constructed with random intercepts for each participant followed by type 2 analysis of variance for measurements with no Wk2 values or using linear mixed-effects models constructed with random intercepts for each participant followed by contrasts of estimated marginal means using the *lme4*, *car* and *modelbased* R packages (31–33). Repeated measures correlations were performed to assess the relationship between dietary components and human milk content using the *rmcorr* R package (34) and were FDR-adjusted. Power analysis determined that n = 13 participants would allow consideration of an effect size greater than 1.6 g/100 mL for HM fat, 248 pg/mL for leptin, 0.16 pg/mL for TNF-α and 92 ng/mL for CRP. Significance was set at alpha ≤0.05. Data analyses were performed using R (version 4.1.0) (35). Extreme outliers were removed if they were 3 times above the upper quartile or 3 times below the lower quartile for all measurements.

## 3 Results

### 3.1 Mother and infant baseline characteristics

Participants were on average 32.8 ± 3.8 years of age and 77% of participants were of non-Hispanic, White descent (Table 1). All participants had obesity at enrollment (mean BMI: 35.9 ± 5.0 kg/m^2^, FM: 46.1 ± 12.3 kg, and FMI: 17.1 ± 4.3 kg/m^2^). Infants showed expected growth with increases in weight and length parameters over the 4-week study (Supplementary Table S3). Of all the measured characteristics, only baseline plasma IL-8 was significantly different between the participants that completed the intervention (2.6 ± 1.1 pg/mL) and those that did not (4.3 ± 1.4 pg/mL).

**Table 1:** Baseline characteristics of lactating women with obesity (*n* = 13) upon enrollment.

	Mean (SD) or *N* (%)
Maternal age (y)	32.8 (3.8)
Maternal height (cm)	163.9 (5.4)
Maternal race	
African American Non-Hispanic	2 (15%)
White Hispanic	1 (8%)
White Non-Hispanic	10 (77%)
Maternal education	
High school/specialized training	3 (23%)
Partial college/college degree	7 (54%)
Graduate Training/Degree	3 (23%)
Household income ($)	
<$40,000	5 (38%)
$40,000 - $70,000	5 (38%)
>$70,000	3 (23%)
Infant sex	
Female	5 (38%)
Male	8 (62%)

### 3.2 Maternal diet quality and metabolic health following dietary intervention

The dietary intervention was started at 4.8 ± 0.28 months postpartum and ended at 5.7 ± months postpartum. The dietary intervention yielded 83.7 and 84.0% compliance to the meal plan at Wk2 and Wk4, respectively. Participants’ HEI increased by 70.5 and 71.0% (+27 and + 27.2 units, *p* < 0.001, respectively) and Mediterranean diet scores increased by 87.1 and 90.6% (+7.4 and + 7.7 units, *p* < 0.001, respectively) whereas daily energy intake was 26.5 and 17.9% and lower (−629 and-424 kcal/d, *p* < 0.001, respectively) at Wk2 and Wk4 of the intervention (Table 2). While on the meal plan, participants’ daily intake was significantly lower for total fat (−37.0% and − 33.0%, *p* < 0.001, respectively), saturated fat (−56.0% and − 52.0%, *p* < 0.001, respectively), polyunsaturated fat (−37.0% and − 34.0%, *p* = 0.01, respectively), ⍵6:⍵3 ratio (−48.3% and − 48.3%, *p* < 0.001), refined grains (−79.0% and − 75.0%, *p* < 0.001, respectively), and sodium (−62.0% and − 54.0%, *p* < 0.001, respectively). Intake of whole grains significantly increased (+60.0% and + 85.0%, *p* < 0.001, respectively). Daily protein intake was significantly different over the duration of the study (Table 2; *p* = 0.029), however, all post-hoc comparisons were non-significant (*p* > 0.05). Daily carbohydrate intake was significantly higher during the intervention at Wk2 compared to pre-intervention (*p* = 0.015).

**Table 2:** Maternal dietary characteristics before (Pre) and during (Wk2 and Wk4) Mediterranean dietary intervention that was provided from 5 to 6 months postpartum to 13 lactating women with obesity.

	Pre	Wk2	Wk4	*p* value
2015 Healthy eating index score	38.3 (5.1)^a^	65.3 (7.6)^b^	65.5 (6.7)^b^	**<0.001**
Compliance to meal plan (%)	-	83.7 (9.7)	84.0 (8.5)	0.96
Mediterranean diet score	8.5 (2.6)^a^	15.9 (3.5)^b^	16.2 (3.2)^b^	**<0.001**
Energy (kcal)	2368.0 (433.1)^a^	1739.3 (299.1)^b^	1944.5 (333.5)^b^	**<0.001**
Total fat (g)	99.6 (23.3)^a^	62.7 (11.2)^b^	66.9 (12.0)^b^	**<0.001**
Total carbohydrates (g)	288.8 (82.9)^a^	220.2 (52.6)^b^	251.5 (62.6)^ab^	**0.009**
Total protein (g)	85.7 (17.1)	84.5 (12.9)	95.6 (10.8)	**0.029**
Sodium (mg)	4055.7 (1008.3)^a^	1542.7 (307.7)^b^	1847.6 (578.8)^b^	**<0.001**
Whole grains (oz. equivalents)	2.0 (1.3)^a^	3.2 (0.9)^b^	3.7 (1.2)^b^	**<0.001**
Refined grains (oz. equivalents)	6.3 (2.6)^a^	1.3 (0.5)^b^	1.6 (0.7)^b^	**<0.001**
Saturated fatty acids (g)	34.5 (10.7)^a^	15.2 (2.3)^b^	16.7 (3.5)^b^	**<0.001**
Polyunsaturated fatty acids (g)	25.4 (9.2)^a^	15.9 (3.9)^b^	16.8 (4.8)^b^	**<0.001**
ω-6 Fatty acids (g)	22.1 (8.2)	12.6 (3.0)	13.1 (4.0)	**<0.001**
ω-3 Fatty acids (g)	2.5 (0.9)	2.8 (0.8)	2.9 (0.9)	0.36
ω-6:ω-3	8.9 (1.6)^a^	4.6 (0.8)^b^	4.6 (1.3)^b^	**<0.001**

Maternal dietary characteristics are summarized as mean (SD). Intervention compliance was calculated as the participants Healthy Eating Index (HEI) score of consumed meals divided by the HEI score of the prescribed meals multiplied by 100. Comparisons were made using linear mixed-effect models followed by contrasting estimated marginal means and values with different superscripts are significantly different. The bolded values are those that were significantly different (*p* < 0.05).

**Figure 1:**
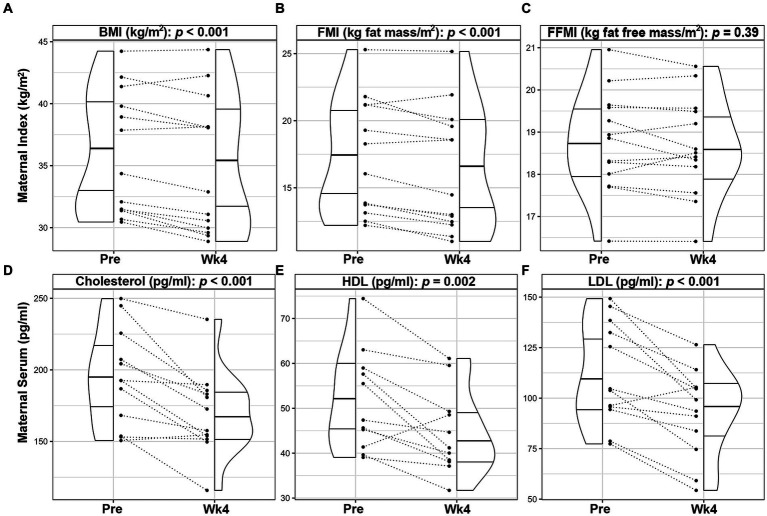
Changes in maternal outcomes of lactating women with obesity following a 4-week Mediterranean dietary intervention. Paired plots showing changes in maternal body mass index [BMI, **(A)**], fat mass index [FMI, **(B)**], fat free mass index [FFMI, **(C)**], cholesterol **(D)**, high density lipoprotein [HDL, **(E)**], and low-density lipoprotein [LDL, **(F)**] between pre-intervention (Pre) and the end of the intervention (Wk4). Dotted lines connect the Pre and Wk4 measures for each participant. Quantiles within the density plots are indicated by the solid horizontal lines.

Following the intervention, maternal weight (−2.7%, *p* < 0.001; Supplementary Table S3), BMI (−2.8%, *p* < 0.001; Figure 1A) and FMI (−5.0%, *p* < 0.001; Figure 1B) significantly decreased by Wk4. FFMI did not change by Wk4 (−0.5%, *p* = 0.39, Figure 1C). Maternal plasma cholesterol levels (total [−12.8%, *p* < 0.001; Figure 1D], HDL [−7.6%, *p* = 0.002; Figure 1E], and LDL [−17.2%, *p* < 0.001; Figure 1F]) were also significantly decreased, even after adjusting for maternal weight loss during the intervention. No significant differences were observed in plasma hormone or cytokine levels between visits (Supplementary Table S3). As expected during the 4-week period, infant length (4.5%, *p* < 0.001), weight (10.6%, *p* < 0.001), fat mass (16.7%, *p* < 0.001), lean mass (9.3%, *p* < 0.001) and FMI (4.3%, *p* < 0.001) increased; however, FFMI, WFA, LFA and WFL did not differ (*p* > 0.05; Supplementary Table S3).

### 3.3 Changes in human milk bioactive molecule concentrations and daily infant intakes

**Figure 2:**
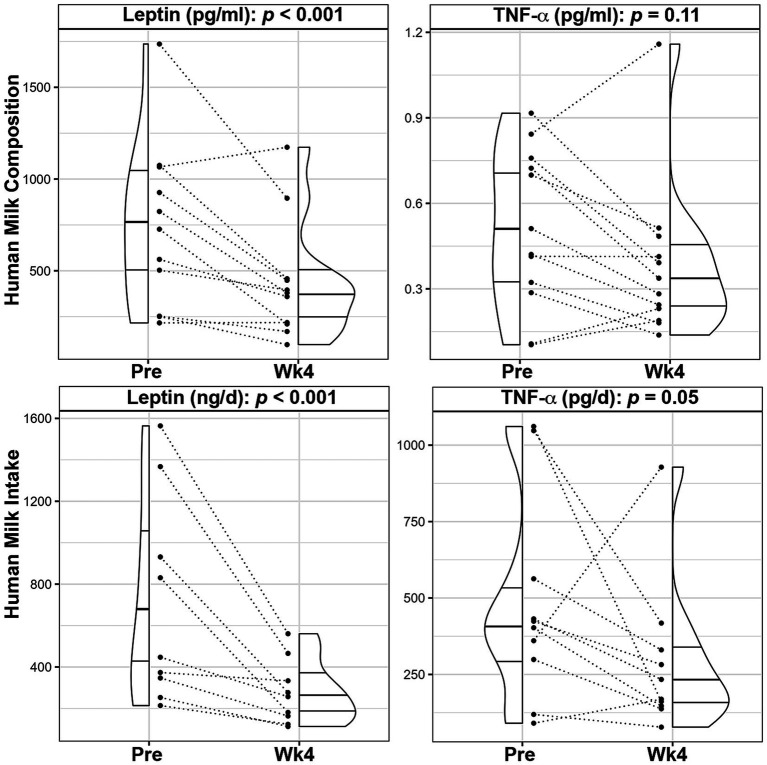
Changes in leptin and TNF-α composition and infant intake following a 4-week Mediterranean dietary intervention. Paired plots showing changes in human milk leptin and tumor necrosis factor α (TNF-α) between pre-intervention (Pre) and the end of the intervention (Wk4). Dotted lines connect the Pre and Wk4 measures for each participant. Quantiles within the density plots are indicated by the solid horizontal lines.

HM collections occurred 5.4 ± 4.5 days before the beginning of the dietary intervention, 0.77 ± 1.5 days before the Wk2 visit and 1.3 ± 2.5 days after finishing the dietary intervention. Following the 4-week intervention, mean HM leptin concentrations significantly decreased by 37.1% (*p* < 0.001, Figure 2; Supplementary Table S4), even after adjusting for maternal weight loss during the intervention. HM total energy (*p* = 0.77) and macronutrient levels (fat: *p* = 0.75, carbohydrate: *p* = 0.60, and protein: *p* = 0.78) did not change, nor did HM concentrations of insulin (*p* = 0.28), CRP (*p* = 0.78), IL-6 (*p* = 0.25), IL-8 (*p* = 0.37), as shown in Supplementary Table S4. HM concentrations of TNF-α (*p* = 0.11) were not significantly different between time points, albeit levels decreased in 9 out of the 13 participants (Figure 2).

**Figure 3:**
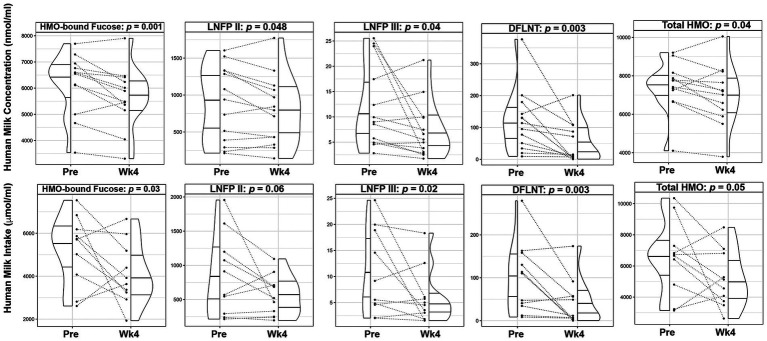
Changes in human milk oligosaccharide composition and infant intake following a 4-week Mediterranean dietary intervention. Paired plots showing changes in composition and intake of human milk oligosaccharide (HMO)-bound fucose, Lacto-N-fucopentaose (LNFP) II, LNFP III, Difucosyllacto-N-tetrose (DFLNT), and Total HMOs between pre-intervention (Pre) and the end of the intervention (Wk4). Dotted lines connect the Pre and Wk4 measures for each participant. Quantiles within the density plots are indicated by the solid horizontal lines.

The mean total HMO concentrations decreased by 6.3% (*p* = 0.036) and mean concentrations of HMO-bound fucose (−8.7%, *p* = 0.001), lacto-N-fucopentaose-II (LNFP II, −10.7%, *p* = 0.048), lacto-N-fucopentaose-III (LNFP III, −38.7%, *p* = 0.041), and difucosyllacto-N-tetrose (DFLNT, −59.4%, *p* = 0.003) were significantly lower following the dietary intervention (Figure 3; Supplementary Table S4). After adjusting for maternal weight loss, concentrations of total HMOs (*p* = 0.075), HMO-bound fucose (*p* = 0.005), LNFP-III (*p* = 0.043), and DFLNT (p = 0.005) remained lower following the intervention.

Mean total HM volume intakes were not significantly different prior to and after the intervention (881.4 ± 310.9 mL vs. 708.0 ± 209.2 mL, *p* = 0.17; Supplementary Table S3). Consistent with lower concentrations, infants’ mean intake of HM leptin (−59.6%, *p* < 0.001) and TNF-α (−38.5%, *p* = 0.05), were significantly lower over the course of the intervention (Figure 2; Supplementary Table S4). Similarly, infants’ mean intake of total HMOs (−24.0%, *p* = 0.05), HMO-bound fucose (−25.5%, *p* = 0.029), LNFP III (−50.4%, *p* = 0.019) and DFLNT (−61.9%, *p* = 0.003) were decreased following the dietary intervention (Figure 3; Supplementary Table S4).

### 3.4 Effect of time on lactation outcomes, a comparison with an observational cohort

To understand the potential impact of time on HM composition and infant intakes, we examined HM parameters and infant intakes from the within-subject study compared with those of an observational cohort of lactating women with obesity at the same months postpartum. There were no demographic differences between the two cohorts (Supplementary Table S5). There were no differences between the studies in HM concentrations of fat, protein, energy, insulin, IL-6 or CRP, or infants’ total milk intake and daily intakes of carbohydrates, protein, energy, insulin, TNF-α, IL-6, or CRP (Supplementary Table S6). There were also no differences in infant weight-for-age, length-for-age or weight-for-length Z-scores or FFMI between cohorts nor did these parameters change with time (Supplementary Table S6). Infant fat mass index was also not different between cohorts, however we did observe that it increased with time, as would be expected in healthy growing infant cohorts.

**Figure 4:**
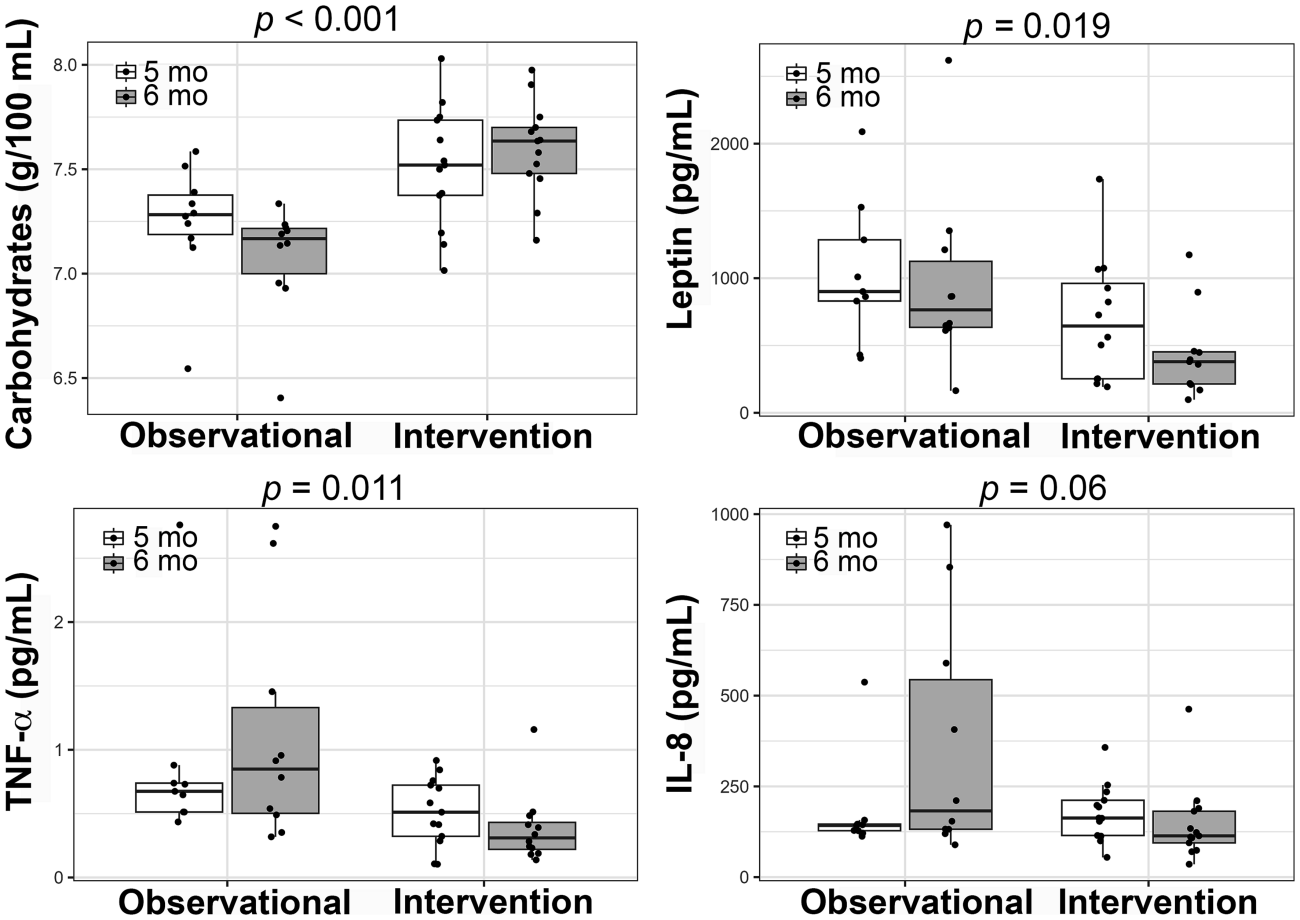
Comparison of changes in human milk composition between the within-subjects intervention and an adjacent, observational cohort. Boxplots showing the changes from 5 to 6 months postpartum in each of the studies for carbohydrate, leptin, tumor necrosis factor-α (TNF-α), and interleukin-8 (IL-8) concentrations. Linear mixed-effects models were used to compare the studies and the *p*-values are presented.

We found that human milk carbohydrate concentrations were higher in the within subject study compared to the observational cohort (5 mo: 7.5 ± 0.29 g/100 mL vs. 7.2 ± 0.29 g/100 mL, 6 mo: 7.6 ± 0.22 g/100 mL vs. 7.1 ± 0.27 g/100 mL; *p* < 0.001) whereas concentrations of human milk leptin (5 mo: 694.4 ± 463.6 pg/mL vs. 1037.8 ± 532.4 pg/mL, 6 mo: 436.7 ± 324.1 pg/mL vs. 963.4 ± 669.5 pg/mL; *p* = 0.019), TNF- α (5 mo: 0.52 ± 0.27 pg/mL vs. 0.88 ± 0.72 pg/mL, 6 mo: 0.38 ± 0.27 pg/mL vs. 1.12 ± 0.89 pg/mL; *p* = 0.011), and IL-8 (trending - 5 mo: 177.7 ± 78.3 pg/mL vs. 176.7 ± 134.7 pg/mL, 6 mo: 146.8 ± 107.1 pg/mL vs. 365.8 ± 328.4 pg/mL; *p* = 0.06) were lower in the within subject study compared to the observation cohort (Figure 4; Supplementary Table S6). Interestingly, we found a significant interaction between time and cohorts for IL-8 HM concentrations (Supplementary Table S6; *p* = 0.012). This was likely a result of the observed increase over time in the observational cohort (delta = 161.3 pg/mL) compared to an observed decrease over time in the within subject cohort (delta = −30.9 pg/mL, Figure 4; Supplementary Table S6).

### 3.5 Association of maternal diet components with human milk composition and daily infant intakes

**Figure 5:**
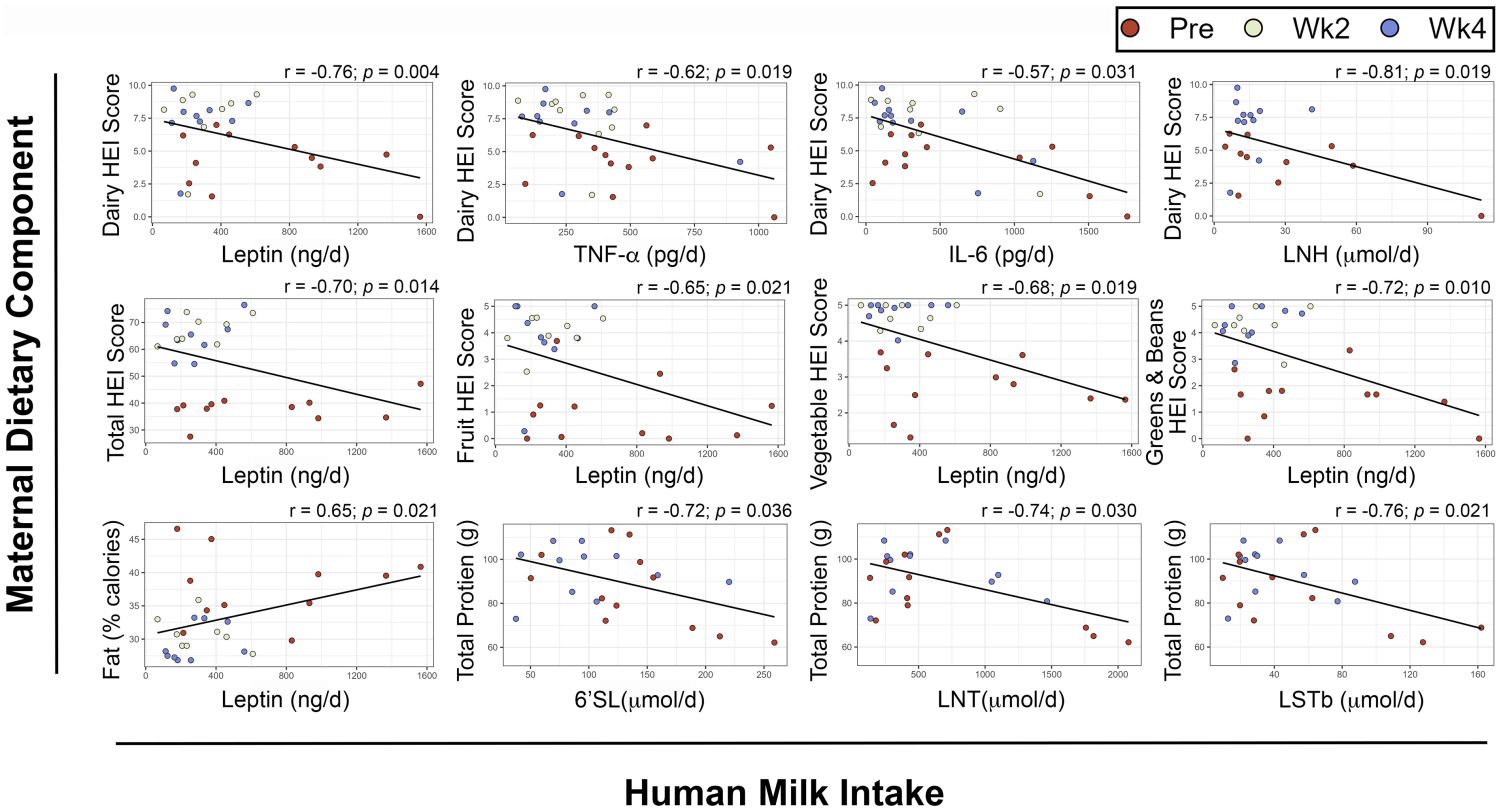
Repeated measures correlations between maternal diet components and infant intake of human milk components. Scatterplots showing relationships between maternal dietary components and infant intakes of human milk components. Maternal dietary intake was analyzed using the Nutrition Data System for Research. Infant intakes were estimated at each visit using test weighing and feeding frequency reported by the mothers. FDR-adjusted p-values are presented. HEI: Healthy Eating Index, 6’SL: 6’Sialyllactose, LNT: Lacto-N-tetrose, LSTb: Sialyl-lacto-N-tetraose b, IL: Interleukin LNH: Lacto-N-hexaose, TNF-α: Tumor Necrosis Factor α.

To determine if specific dietary components had a direct relationship with HM or infant outcomes, we performed repeated measures correlations (Figure 5). After FDR-adjustment, no components of maternal diet were associated with HM composition. Several dietary components showed significant, negative associations with infant daily intake of leptin (Figure 5) including maternal dairy HEI score (r_rm_ = −0.76, *p*_rm adjusted_ = 0.004), total HEI score (r_rm_ = −0.70, *p*_rm adjusted_ = 0.014), total fruit HEI score (r_rm_ = −0.65, *p*_rm adjusted_ = 0.021), total vegetable HEI score (r_rm_ = −0.68, *p*_rm adjusted_ = 0.019), and greens and beans HEI score (r_rm_ = −0.72, *p*_rm adjusted_ = 0.01). Conversely, maternal fat intake as a percentage of daily calories was positively associated with infant daily intake of leptin (r_rm_ = 0.65, *p*_rm adjusted_ = 0.021).

There were also negative associations between maternal dairy HEI score and infant intakes of TNF-α (r_rm_ = −0.62, *p*_rm adjusted_ = 0.019), IL-6 (r_rm_ = −0.57, *p*_rm adjusted_ = 0.031), and Lacto-N-hexaose (LNH, r_rm_ = −0.81, *p*_rm adjusted_ = 0.019).

Maternal total protein intake was negatively associated with infant intakes of the HMOs 6′Sialyllactose (6’SL, r_rm_ = −0.72, *p*_rm adjusted_ = 0.036), Lacto-N-tetrose (LNT, r_rm_ = −0.74, *p*_rm adjusted_ = 0.03) and Sialyl-lacto-N-tetraose b (LSTb, r_rm_ = −0.76, *p*_rm adjusted_ = 0.021).

Adjusted linear mixed effects models were used to investigate the relationships between HEI score components and infant intakes while adjusting for total HEI score (Table 3). The adjusted β coefficients were less than 25% different for the following models: dairy HEI score and daily infant intake of TNF-α (14.5%), and greens and beans HEI score and daily infant intake of leptin (16.5%). Together, these data suggest that the observed relationship between dietary components of maternal HEI scores and infant intakes of human milk components were not dependent on total HEI scores.

**Table 3:** Summaries of raw and adjusted Healthy Eating Index models.

HEI Component	Infant Intakes	Raw ß	Adjusted ß^*^	% Difference between raw and adjusted ß
Dairy HEI score	IL-6 (pg/d)	−105.17	−134.65	28.04
Dairy HEI score	Leptin (ng/d)	−85.22	−60.17	29.39
Dairy HEI score	LNH (μmol/d)	−5.84	−7.50	28.39
Dairy HEI score	TNF-α (pg/d)	−50.60	−43.27	14.48
Greens and beans HEI score	Leptin (ng/d)	−122.91	−102.67	16.47
Total vegetable HEI score	Leptin (ng/d)	−160.83	−117.79	26.76
Total fruit HEI score	Leptin (ng/d)	−95.95	−40.88	57.39

HEI, Healthy Eating Index; IL, Interleukin; LNH, Lacto-N-hexaose; TNF-α, Tumor Necrosis Factor α. The bolded values are those that had % differences less than 25%. ^*^Model adjusted for Total HEI Score.

## 4 Discussion

The growing evidence that having overweight and obesity modulates HM composition in ways that can promote infant adiposity (2, 4, 5, 9) warrants the development of interventions that may temper these effects. In this study, we tested the effect of a 4-week Mediterranean meal plan, implemented at 5 months postpartum in women with obesity and demonstrated for the first time that the intervention improved maternal HEI scores and plasma lipid profiles, reduced maternal BMI and fat mass, decreased HM concentrations and infants’ intakes of leptin, TNF-α, LNFP II, LNFP-III, DFLNT, total HMOs and HMO-bound fucose. While we observed differences in the concentrations of HM leptin, maternal circulating levels of leptin did not change, indicating other potential avenues for maternal dietary interventions to alter HM composition such as altering leptin production locally in the mammary gland (36). Additionally, we identified individual dietary components (e.g., protein, fat) and HEI score components (e.g., dairy, total fruit) that were significantly associated with intakes of bioactive molecules in HM. These findings provide compelling evidence that dietary interventions during lactation can mitigate obesity-associated alterations in HM composition that may ultimately affect early-life nutritional programing of infant health while promoting maternal health.

### 4.1 Maternal diet and maternal outcomes

In non-pregnant/non-lactating women with obesity, adherence to a Mediterranean diet has been shown to decrease BMI (16, 17), circulating obesogenic hormones (17), adipokines (18), and systemic inflammation (16, 19). Findings from this study suggest that similar results can be attained in lactating women with obesity, which is of importance to prevent postpartum weight retention and optimize maternal and child health (37). Several randomized controlled trials have demonstrated the efficacy of caloric restriction and/or exercise on weight loss and body composition during the postnatal period, although they failed to evaluate their impact on HM composition or infant health (38). By proxy, replacing habitual post-partum maternal diet with a Mediterranean dietary pattern in our study population resulted in a reduced caloric intake while meeting dietary guideline recommendations. Consistent with our findings, caloric restriction resulted in significant weight loss and improved body composition that was sustained for up to 1 year in some studies (38, 39). Stendell-Hollis et al. also demonstrated that 4 months of a Mediterranean diet or a MyPyramid diet were effective in reducing postpartum maternal weight, fat mass, and plasma TNF-α levels (40). Low-fat diets, and diets that are high in fiber decreased HDL levels in adults with normal weight and overweight/obesity, similar to what we observed in lactating women (41, 42). Future studies will need to elucidate the unique contributions of energy deficit vs. maternal dietary quality to changes in HM composition and their benefits to the child.

### 4.2 Maternal diet and human milk bioactives

Women with obesity have elevated pro-inflammatory chemokines (2, 11), leptin (2, 3), and insulin (2, 8) HM content compared to peers with normal weight. Importantly, infants’ intakes of HM insulin and CRP were significantly and positively associated with their fat mass index (2). It is believed that obesity-related systemic and local (mammary gland microenvironment) inflammation (43) may contribute to elevations in pro-inflammatory cytokines that have been observed in HM from women with obesity (2, 11). While much research has focused on how maternal obesity influences bioactives in HM (10), few studies exist describing the relationships between maternal dietary intake and HM bioactives. In animal models of obesity, caloric restriction has led to decreases in mammary gland inflammation (43, 44). Our study expands on these findings and demonstrates a reduction in HM pro-inflammatory cytokines (TNF-α and IL-8) from women with obesity who underwent a Mediterranean dietary intervention. In agreement with our current data, improved maternal dietary quality and reduced caloric intake led to lower HM insulin and leptin levels (17). Critically, infant intakes of these HM components were also reduced following the 4-week intervention. In accordance with our study, a previous investigation reported reduced caloric intake for 2 weeks did not result in changes in concentrations or infant intakes of HM macronutrients or in changes in infant weight-for-length, weight-for-age, or length-for-age z-scores, despite changes in infant intakes of leptin and insulin (15). With such an acute intervention, changes in infant growth that could be attributed to HM components would not necessarily be expected. Therefore, future studies that can implement dietary interventions throughout the postnatal period are critical to understanding the potential positive impact such dietary interventions may have on HM composition and subsequent offspring body composition.

We and others have also shown positive associations between maternal obesity and HMO content (4, 45). These associations are important because HMOs are among the most predominant bioactive components in HM, supporting infant gut development (46, 47) and the prevention of infectious diseases (47). However, recent evidence also suggests that some HMOs that are elevated in HM from women with obesity are associated with infant growth (greater weight-for-length Z-scores) and fat accretion (4, 48, 49). While LNFP III was not associated with maternal obesity in our previous study (4), it showed a strong positive association with infant fat mass at 2 months of age (4). This is important because in the current study, HM concentrations and infant intakes of LNFP III were significantly lower following the dietary intervention. Similarly, previous studies have found significant positive associations between HMO concentrations (disialyllacto-N-tetraose, LNFP II, total HMO concentrations, and total HMO-bound fucose) and infant fat mass at 5–6 months of age (48, 49), many of which were significantly reduced following the Mediterranean diet intervention in this study. It is important to recognize that HMO concentrations change over lactation (50, 51) and change in relation to maternal BMI (4). While these pilot data present compelling evidence for dietary influences on HMOs, our study did not include a prospective control group to test sufficiently the effect of dietary intervention vs. time on HMO concentrations nor did we have time-matched retrospective data on HMO concentrations in our observational cohort.

Previous studies have shown that maternal consumption of fruit, whole grains, and specific fatty acids have been associated with individual HMO concentrations (51–53). Our data are not completely aligned with these previous reports. It is possible that these discrepancies are related to differences in the analyses of dietary intake data (e.g., food frequency questionnaires vs. daily food records), in the timing of sample collection, or in the analytical approaches of the studies. Comparable to our data, Azad et al. reported no significant association between maternal HEI score and HMO concentrations (51). However, Azad et al. did find a weak but significant negative association between maternal total protein intake and LSTb concentrations consistent with the infant intake data presented herein. LSTb showed a strong, positive association with infant fat mass as well as weight-for-length and weight for age Z-scores in our previous study (4), suggesting that maternal protein intake may be a modifiable factor that can be used in future intervention studies to improve infant body composition. A recent short-term crossover study employing four different dietary paradigms (galactose vs. glucose and high carbohydrate vs. high fat) demonstrated a significant association between maternal dietary energy source and the concentrations of HMOs (14) further supporting the notion that interventions focused on specific dietary components may benefit infant health through alterations to HMOs.

### 4.3 Limitations and strengths

Caution should be taken when interpreting these results because of the small sample size of mainly non-Hispanic White lactating women and the lack of a concurrent control group for comparison. Yet, given the US population-wide exclusive breastfeeding rates at 6 months of 24.9% (54), this study assesses the feasibility of Mediterranean diet pattern implementation in an exclusively breastfeeding cohort of women with obesity. Therefore, reporting data from 13 participants of this population provides foundational knowledge for future Mediterranean diet intervention designs. There is a clear need to conduct randomized control trials to confirm our pilot-study findings and to use standardized methodology to increase reproducibility and rigor of future research. A second limitation is the confounding effect of calorie restriction that occurred by replacing habitual dietary patterns with the Mediterranean diet plan in this study. While this prevents us from exclusively attributing the assessed effects to the change in diet quality, it provides insights to the prevailing nutrient poor, calorie dense habitual diets in the assessed cohort. Furthermore, participants consumed an average of 1841 kcal/d during the intervention, which is in-line with DGA for sedentary women ages 19-50y, as an additional 450–500 kcal/d intake during breastfeeding is only recommended for women aiming to maintain post-partum weight (22). Third, the effects of storage at 4°C of the HM during the 24-h collection at the participants’ homes were not investigated. Despite these limitations, this pilot study provides unique results that healthy dietary habits can influence maternal health, HM composition, and children’s HM intakes during the postpartum period in women with obesity. There were several significant strengths to this study, including: (1) greater than 80% adherence to the dietary intervention that resulted in significant improvements in maternal diet quality and body composition in only 4 weeks, (2) significant changes in human milk bioactive components and (3) measuring infant HM intakes and acquiring representative milk samples over 24-h to use best practices in estimating infants’ exposures. Future studies will need to evaluate a more diverse population, larger cohort, and a longer length of intervention while maintaining isocaloric intakes and body weight from baseline.

### 4.4 Conclusion

This study is the first to demonstrate the feasibility of implementing a Mediterranean meal plan in lactating women with obesity while examining its impact on human milk composition, infant intake, and infant anthropometrics. This in-depth investigation allows for a better understanding of the dynamic of the breastfeeding triad of mother/milk/infant and how a healthy diet could improve maternal and child health.

## Data availability statement

The raw data supporting the conclusions of this article will be made available by the authors, without undue reservation.

## Ethics statement

The studies involving humans were approved by University of Arkansas for Medical Sciences Institutional Review Board. The studies were conducted in accordance with the local legislation and institutional requirements. Written informed consent for participation in this study was provided by the participant.

## Author contributions

CS: Writing – review & editing, Writing – original draft, Visualization, Project administration, Investigation, Funding acquisition, Formal analysis, Conceptualization. JLS: Writing – review & editing, Writing – original draft. AM: Writing – review & editing, Investigation, Formal analysis, Conceptualization. SS: Writing – review & editing, Writing – original draft. MC: Writing – review & editing, Investigation. JB: Writing – review & editing, Investigation, Formal analysis. DT: Writing – review & editing, Investigation, Formal analysis. AF: Writing – review & editing, Investigation. LJ: Writing – review & editing, Writing – original draft. LB: Writing – review & editing, Investigation, Conceptualization. AA: Writing – review & editing, Writing – original draft, Supervision, Project administration, Investigation, Funding acquisition, Conceptualization.

## Glossary

6’SL: 6’Sialyllactose
BMI: Body mass index
CRP: C-reactive protein
DFLNT: Difucosyllacto-N-tetrose
FFM: Fat free mass
FFMI: Fat free mass index
FM: Fat mass
FMI: Fat mass index
HDL: High density lipoprotein
HEI: Healthy eating index
HM: Human milk
HMO: Human milk oligosaccharides
IL: Interleukin
LDL: Low density lipoprotein
LNFP: Lacto-N-fucopentaose
LNH: Lacto-N-hexaose
LNT: Lacto-N-tetrose
LSTb: Sialyl-lacto-N-tetraose b
TNF-α: Tumor necrosis factor-α
Wk: Week
