# Supplementary material for: A Mediterranean diet plan in lactating women with obesity reduces maternal energy intake and modulates human milk composition – a feasibility study
Source: Front Nutr. 2024 Mar 13;11:1303822. doi: 10.3389/fnut.2024.1303822 (PMC10965561; doi:10.3389/fnut.2024.1303822)
Supplement: Supplementary file 1 [file Table_1.DOCX]

Supplementary Material


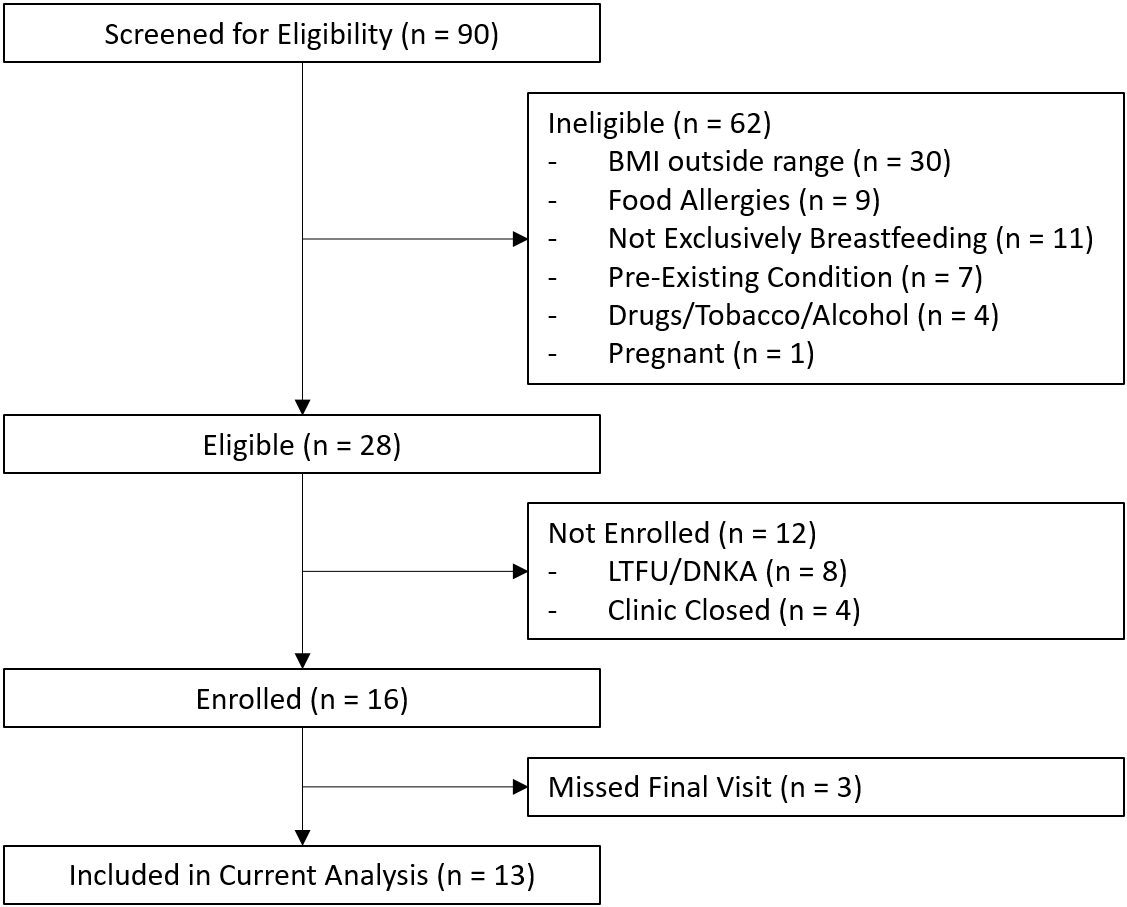


**Supplementary Figure 1.** **Cohort flow diagram**. Diagram showing the number of participants screened, eligible and enrolled in the study and the number of participants that completed the study and are included in the current analysis. BMI: Body Mass Index; LTFU: Lost to Follow Up; DNKA: Did Not Keep Appointment.

**Supplementary Table 1:** Nutrition Information of Prescribed Diet

| **Dietary Component** | **Daily Intake** |
| --- | --- |
| Energy (kcal) | 2,061.29 |
| %kcal from Fat | 27.78 |
| %kcal from Carbohydrates | 52.58 |
| %kcal from Protein | 18.89 |
| Fat (g) | 66.06 |
| Saturated Fat (g) | 14.43 |
| Monounsaturated Fat (g) | 34.41 |
| Polyunsaturated Fat (g) | 17.60 |
| Polyunsaturated Fat : Saturated Fat | 1.19 |
| Trans Fatty Acids (g) | 0.51 |
| Carbohydrates (g) | 276.95 |
| Grains (oz/eq) | 5.38 |
| Whole Grains (oz/eq) | 4.38 |
| Refined Grains (oz/eq) | 1.01 |
| Protein (g) | 100.52 |
| Animal Protein (g) | 66.42 |
| Vegetable Protein (g) | 37.01 |
| Sodium (mg) | 1,270.04 |
| 2015 Healthy Eating Index Score (HEI) | 77.99 |
| *Note:* Nutrition Information was determined by analyzing the prescribed diet using the Nutrition Data System for Research (NDSR) program. | |

**Supplementary Table 2:** Example Weekly Menu of Prescribed Diet

|  | **Breakfast** | **Lunch** | **Dinner** | **Snack** |
| --- | --- | --- | --- | --- |
| **Monday** | English Muffin Sandwich (1) Smoothie (3/4 c frozen fruit, 1 c milk) | Greek Pasta Bowl (1 cont) Zucchini (1 cup) Olive Oil (1 Tb) | Turkey Patty Meal (1 cont) Sweet Potato (1 cup) Olive Oil (1 Tb) | Canned Fruit (1 can)  Walnuts (1/4 cup) 1% Milk (1.5 cups) |
| **Tuesday** | Oatmeal (1 cup - made with 2 cups 1% milk) Frozen Fruit (3/4 cup) | Basa Meal (1 cont) Cauliflower (1 cup) Olive Oil (1 Tb) | Chicken Tenders Meal (1 cont) Yellow Squash (1 cup) Olive Oil (1 Tb) | Canned Fruit (1 can) Greek Yogurt (1 cont) |
| **Wednesday** | English Muffin Sandwich (1) Smoothie (3/4 c frozen fruit, 1 c milk) | Bean Chili (1 cont) Broccoli (1 cup) Olive Oil (1 Tb) | Salmon Meal (1 cont) Sweet Potato (1 cup) Olive Oil (1 Tb) | Canned Fruit (1 can)  Walnuts (1/4 cup) 1% Milk (1.5 cups) |
| **Thursday** | Oatmeal (1 cup - made with 2 cups 1% milk) Frozen Fruit (3/4 cup) | Beef Patty Meal (1 cont) Sweet Potato (1 cup) Olive Oil (1 Tb) | BBQ Veg Lentils (1 cont) Zucchini (1 cup) Olive Oil (1 Tb) | Canned Fruit (1 can) Greek Yogurt (1 cont) |
| **Friday** | English Muffin Sandwich (1) Smoothie (3/4 c frozen fruit, 1 c milk) | Chicken Tenders Meal (1 cont) Yellow Squash (1 cup) Olive Oil (1 Tb) | Turkey Patty Meal (1 cont) Cauliflower (1 cup) Olive Oil (1 Tb) | Canned Fruit (1 can)  Walnuts (1/4 cup) 1% Milk (1.5 cups) |
| **Saturday** | Oatmeal (1 cup - made with 2 cups 1% milk) Frozen Fruit (3/4 cup) | Thyme White Bean and Yams (1 cont) Broccoli (1 cup) Olive Oil (1 Tb) | Basa Meal (1 cont) Sweet Potato (1 cup) Olive Oil (1 Tb) | Canned Fruit (1 can) Greek Yogurt (1 cont) |
| **Sunday** | Oatmeal (1 cup - made with 2 cups 1% milk) Frozen Fruit (3/4 cup) | Salmon Meal (1 cont) Zucchini (1 cup) Olive Oil (1 Tb) | Olive and Pinto Bean Quinoa (1 cont) Yellow Squash (1 cup) Olive Oil (1 Tb) | Canned Fruit (1 can) Greek Yogurt (1 cont) |

**Supplementary Table 3:** Maternal and Infant Characteristics

|  | **Pre** | **Wk2** | **Wk4** | ***p* value** |
| --- | --- | --- | --- | --- |
| **Maternal Characteristics** | | | | |
| Weight (kg) | 96.6 (15.2)^a^ | 94.7 (15.6)^b^ | 94.0 (16.1)^b^ | **< 0.001** |
| BMI (kg/m^2^) | 35.9 (5.0)^a^ | 35.2 (5.2)^b^ | 34.9 (5.5)^b^ | **< 0.001** |
| Fat Mass (kg) | 46.1 (12.3)^a^ | 44.5 (12.8)^b^ | 43.8 (12.9)^b^ | **< 0.001** |
| Fat Free Mass (kg) | 50.5 (4.4) | 50.2 (4.2) | 50.2 (4.4) | 0.39 |
| Fat Mass Index (kg fat mass/m^2^) | 17.1 (4.3)^a^ | 16.5 (4.5)^b^ | 16.3 (4.6)^b^ | **< 0.001** |
| Fat Free Mass Index (kg fat free mass/m^2^) | 18.8 (1.2) | 18.7 (1.1) | 18.7 (1.2) | 0.39 |
| Cholesterol (mg/dl) | 194.1 (34.2) | ̶ | 169.2 (29.4) | **< 0.001** |
| HDL (mg/dl) | 51.6 (11.2) | ̶ | 47.7 (14.1) | **0.002** |
| LDL (mg/dl) | 111.8 (25.3) | ̶ | 92.6 (21.5) | **< 0.001** |
| Triglycerides (mg/dl) | 89.0 (29.6) | ̶ | 94.5 (37.8) | 0.49 |
| Insulin (mg/dl) | 300.7 (163.2) | ̶ | 262.4 (146.1) | 0.09 |
| Leptin (ng/ml) | 23.6 (11.2) | ̶ | 20.8 (16.6) | 0.38 |
| CRP (µg/ml) | 6.8 (4.8) | ̶ | 5.5 (4.5) | 0.28 |
| IL-6 (pg/ml) | 0.6 (0.3) | ̶ | 0.7 (0.3) | 0.36 |
| IL-8 (pg/ml) | 2.6 (1.2) | ̶ | 3.1 (1.3) | 0.26 |
| TNF-α (pg/ml) | 1.7 (0.3) | ̶ | 1.6 (0.2) | 0.60 |
| **Infant Characteristics** | | | | |
| Total Milk Intake (ml) | 881.4 (310.9) | 686.2 (290.5) | 708.0 (209.2) | 0.17 |
| Length (cm) | 62.2 (2.0)^a^ | 63.8 (1.9)^b^ | 65.0 (1.8)^c^ | **< 0.001** |
| Weight (kg) | 6.6 (0.7)^a^ | 7.0 (0.6)^b^ | 7.3 (0.7)^c^ | **< 0.001** |
| Fat Mass (kg) | 1.8 (0.5) | 2.0 (0.4) | 2.1 (0.4) | **< 0.001** |
| Lean Mass (kg | 4.3 (0.5) | 4.5 (0.5) | 4.7 (0.5) | **< 0.001** |
| Fat Mass Index (kg fat mass/m^2^) | 4.7 (1.4) | 4.8 (0.9) | 4.9 (0.9) | **< 0.001** |
| Fat Free Mass Index (kg fat free mass/m^2^) | 11.1 (0.6) | 11.0 (0.7) | 11.0 (0.8) | 0.48 |
| Weight-for-Age Z-Score | -0.5 (0.8) | -0.5 (0.7) | -0.4 (0.7) | 0.58 |
| Length-for-Age Z-Score | -0.9 (0.9) | -0.8 (0.7) | -0.7 (0.7) | 0.30 |
| Weight-for-Length Z-Score | 0.2 (0.8) | 0.1 (0.7) | 0.1 (0.8) | 0.51 |
| *Note:* Maternal and infant clinical and demographic characteristics are summarized as mean ± SD. Comparisons were made using linear mixed-effect models followed by ANOVA. For comparisons across all three visits, comparisons were made contrasting estimated marginal means and values with different superscripts are significantly different. The bolded values are those that were significantly different (p < 0.05). | | | | |

**Supplementary Table 4:** Human Milk Composition and Infant Intakes

|  | **Human Milk Composition** | | | | **Infant Intake** | | | |
| --- | --- | --- | --- | --- | --- | --- | --- | --- |
|  | **Pre** | **Wk2** | **Wk4** | ***p* value** | **Pre** | **Wk2** | **Wk4** | ***p* value** |
| Fat (g/100 ml and g/d) | 4.9 (1.6) | 5.0 (1.6) | 4.7 (1.6) | 0.75 | 39.8 (14.4) | 33.3 (17.3) | 30.2 (11.1) | 0.22 |
| Carbohydrates (g/100 ml and g/d) | 7.5 (0.3) | 7.5 (0.2) | 7.6 (0.2) | 0.60 | 67.0 (25.6) | 51.3 (21.2) | 53.9 (17.0) | 0.16 |
| Protein (g/100 ml and g/d) | 0.8 (0.2) | 0.7 (0.1) | 0.7 (0.2) | 0.78 | 6.6 (2.5) | 4.8 (1.8) | 5.5 (2.0) | 0.13 |
| Energy (kcal/100 ml and kcal/d) | 79.8 (14.4) | 80.7 (14.9) | 77.7 (13.7) | 0.77 | 673.1 (191.8) | 540.1 (228.0) | 526.9 (144.1) | 0.12 |
| Insulin (pg/ml and ng/d)) | 1297.4 (830.5) | 970.2 (485.8) | 1145.8 (623.7) | 0.28 | 1002.9 (498.2) | 599.6 (313.6) | 770.7 (478.2) | 0.08 |
| Leptin (pg/ml and ng/d)) | 694.4 (463.6)^a^ | 533.6 (374.8)^b^ | 436.7 (324.1)^b^ | **< 0.001** | 680.8 (483.7)^a^ | 295.5 (167.6)^b^ | 275.0 (154.9)^b^ | **< 0.001** |
| CRP (ng/ml and µg/d) | 156.3 (194.4) | 144.0 (155.8) | 130.2 (153.5) | 0.78 | 111.4 (117.2) | 100.7 (120.6) | 98.6 (141.5) | 0.85 |
| IL-6 (pg/ml and pg/d) | 0.7 (0.7) | 0.7 (0.7) | 0.5 (0.5) | 0.25 | 626.4 (595.4) | 425.5 (380.6) | 338.7 (348.9) | 0.11 |
| IL-8 (pg/ml and ng/d) | 177.7 (78.3) | 170.8 (89.2) | 146.8 (107.1) | 0.37 | 153.5 (56.9) | 104.4 (46.2) | 99.5 (95.4) | 0.09 |
| TNF-α (pg/ml and pg/d) | 0.5 (0.3) | 0.5 (0.2) | 0.4 (0.3) | 0.11 | 490.0 (304.1)^a^ | 301.3 (117.9)^a^ | 288.4 (246.8)^a^ | **0.05** |
| 2’FL (nmol/ml and µmol/d) | 1753.4 (2038.9) | ̶ | 1906.3 (2601.1) | 0.59 | 1546.4 (1810.4) | ̶ | 1725.1 (2152.5) | 0.82 |
| 3FL (nmol/ml and µmol/d) | 2671.3 (1715.8) | ̶ | 2626.0 (1476.5) | 0.72 | 2306.4 (1769.7) | ̶ | 1617.5 (977.9) | 0.17 |
| 3’-SL (nmol/ml and µmol/d) | 234.6 (162.3) | ̶ | 227.2 (154.9) | 0.68 | 204.4 (131.3) | ̶ | 184.2 (143.1) | 0.43 |
| DFLac (nmol/ml and µmol/d) | 35.2 (38.5) | ̶ | 31.8 (41.5) | 0.61 | 31.7 (37.8) | ̶ | 27.0 (33.9) | 0.44 |
| 6’-SL (nmol/ml and µmol/d) | 148.5 (44.6) | ̶ | 131.8 (87.2) | 0.36 | 139.3 (59.3) | ̶ | 100.8 (52.5) | 0.06 |
| LNT (nmol/ml and µmol/d) | 758.8 (528.2) | ̶ | 738.6 (534.9) | 0.75 | 770.6 (695.7) | ̶ | 583.8 (436.1) | 0.27 |
| LNnT (nmol/ml and µmol/d) | 56.4 (32.4) | ̶ | 51.9 (35.9) | 0.19 | 53.0 (44.7) | ̶ | 31.5 (18.5) | 0.08 |
| LNFP I (nmol/ml and µmol/d) | 345.8 (331.6) | ̶ | 366.7 (369.2) | 0.51 | 320.4 (277.2) | ̶ | 312.8 (283.5) | 0.64 |
| LNFP II (nmol/ml and µmol/d) | 882.3 (513.1) | ̶ | 787.6 (468.7) | **0.048** | 863.6 (610.8) | ̶ | 553.7 (292.1) | 0.06 |
| LNFP III (nmol/ml and µmol/d) | 11.9 (8.3) | ̶ | 7.3 (5.6) | **0.041** | 11.7 (8.4) | ̶ | 5.8 (5.2) | **0.019** |
| LSTb (nmol/ml and µmol/d) | 59.0 (40.6) | ̶ | 54.5 (35.6) | 0.43 | 59.8 (48.9) | ̶ | 39.1 (24.7) | 0.07 |
| LSTc (nmol/ml and µmol/d) | 21.2 (12.7) | ̶ | 23.2 (19.7) | 0.87 | 18.0 (12.2) | ̶ | 16.8 (11.3) | 0.73 |
| DFLNT (nmol/ml and µmol/d) | 120.0 (97.4) | ̶ | 48.7 (62.2) | **0.003** | 109.4 (85.0) | ̶ | 41.7 (53.4) | **0.003** |
| LNH (nmol/ml and µmol/d) | 27.5 (20.2) | ̶ | 22.3 (10.0) | 0.41 | 30.8 (32.4) | ̶ | 15.6 (9.4) | 0.14 |
| DSLNT (nmol/ml and µmol/d) | 65.5 (38.7) | ̶ | 76.1 (47.8) | 0.13 | 63.8 (51.8) | ̶ | 59.2 (44.9) | 0.75 |
| FLNH (nmol/ml and µmol/d) | 80.4 (38.7) | ̶ | 83.0 (58.1) | 0.92 | 79.4 (48.9) | ̶ | 63.4 (32.9) | 0.29 |
| DFLNH (nmol/ml and µmol/d) | 21.3 (18.7) | ̶ | 19.8 (15.3) | 0.74 | 16.8 (16.5) | ̶ | 16.6 (14.4) | 0.86 |
| FDSLNH (nmol/ml and µmol/d) | 66.5 (44.5) | ̶ | 66.3 (38.5) | 0.98 | 63.0 (45.4) | ̶ | 49.5 (26.5) | 0.25 |
| DSLNH (nmol/ml and µmol/d) | 39.6 (15.6) | ̶ | 33.8 (28.9) | 0.44 | 35.5 (15.8) | ̶ | 25.9 (18.1) | 0.09 |
| Total HMOs (nmol/ml and µmol/d) | 7441.4 (1260.4) | ̶ | 6974.8 (1583.0) | **0.036** | 6746.7 (2256.8) | ̶ | 5128.3 (1834.1) | **0.05** |
| HMO-bound Sialylate (nmol/ml and µmol/d) | 806.6 (278.4) | ̶ | 861.8 (332.6) | 0.42 | 746.3 (362.3) | ̶ | 645.0 (278.9) | 0.28 |
| HMO-bound Fucose (nmol/ml and µmol/d) | 6184.4 (1145.2) | ̶ | 5644.3 (1180.9) | **0.001** | 5521.0 (1653.3) | ̶ | 4123.2 (1450.7) | **0.029** |
| Milk Intake per Nursing Session (ml) | ̶ | ̶ | ̶ | ̶ | 128.0 (41.2) | 98.9 (47.8) | 111.0 (50.6) | 0.08 |
| \| *Note*: Human milk component concentrations and infant intakes are summarized as mean ± SD. Comparisons were made using linear mixed-effect models followed by ANOVA. For comparisons across all three visits, values with different superscripts are significantly different. The bolded values are those that were significantly different (p < 0.05). \| \| --- \| | | | | | | | | |

**Supplementary Table 5**: Demographics of Observational and Intervention Cohorts

|  | **Observational (N=10)** | **Intervention (N=13)** | ***P* value** |
| --- | --- | --- | --- |
| **Maternal Age (years)** | 31.9 (3.2) | 32.8 (3.8) | 0.320 |
| **Maternal BMI (kg/m^2^)** | 33.15 (2.29) | 35.86 (5.00) | 0.321 |
| **Maternal Race** |  |  | 0.772 |
| African American | 2 (20.0%) | 2 (15.4%) |  |
| White | 8 (80.0%) | 11 (84.6%) |  |
| **Maternal Ethnicity** |  |  | 0.370 |
| Hispanic | 0 (0.0%) | 1 (7.7%) |  |
| Non-Hispanic | 10 (100.0%) | 12 (92.3%) |  |
| **Infant Sex** |  |  | 0.940 |
| Female | 4 (40.0%) | 5 (38.5%) |  |
| Male | 6 (60.0%) | 8 (61.5%) |  |

**Supplementary Table 6**: Comparison of human milk composition and infant intakes in observational cohort and intervention cohort.

|  | **Observational Cohort** | | | | **Intervention Cohort** | | | | ***p* value** | | |
| --- | --- | --- | --- | --- | --- | --- | --- | --- | --- | --- | --- |
|  | **5 mo** | **6 mo** | **Mean Δ** | ***p* value** | **5 mo** | **6 mo** | **Mean Δ** | ***p* value** | **Value Differs over Time** | **Value Differs between Cohorts** | **Interaction between Time and Cohort** |
| Total Milk Intake (ml) | 761.9 | 824.4 | 145.2 | 0.69 | 881.4 | 708.0 | -145.1 | 0.17 | 0.57 | 0.93 | 0.30 |
| Fat Concentration (g/100 ml) | 4.4 | 5.4 | 1.0 | 0.28 | 4.9 | 4.7 | -0.2 | 0.41 | 0.54 | 0.88 | 0.19 |
| Fat Intake (g/d) | 28.2 | 44.2 | 13.1 | 0.44 | 39.8 | 30.2 | -8.2 | 0.07 | 0.97 | 0.80 | **0.037** |
| Carbohydrate Concentration (g/100 ml) | 7.2 | 7.1 | -0.2 | 0.13 | 7.5 | 7.6 | 0.1 | 0.59 | 0.75 | **< 0.001** | 0.10 |
| Carbohydrate Intake (g/d) | 56.7 | 62.5 | -3.9 | 1.00 | 67.0 | 53.9 | -10.8 | 0.37 | 0.43 | 0.98 | 0.36 |
| Protein Concentration (g/100 ml) | 0.8 | 0.7 | 0.0 | 0.32 | 0.8 | 0.7 | 0.0 | 0.65 | 0.36 | 0.75 | 0.86 |
| Protein Intake (g/d) | 6.2 | 6.6 | -0.7 | 0.84 | 6.6 | 5.5 | -0.8 | 0.58 | 0.46 | 0.76 | 0.59 |
| Energy Concentration (kcal/100 ml) | 74.2 | 82.1 | 7.9 | 0.28 | 79.8 | 77.7 | -2.1 | 0.41 | 0.58 | 0.91 | 0.22 |
| Energy Intake (kcal/d) | 522.6 | 694.9 | 101.2 | 0.56 | 673.1 | 526.9 | -124.0 | 0.07 | 0.69 | 0.86 | 0.06 |
| Leptin Concentration (pg/ml) | 1,037.8 | 963.4 | -117.7 | 0.30 | 694.4 | 436.7 | -303.3 | **0.007** | **0.029** | **0.019** | 0.33 |
| Leptin Intake (ng/d) | 1,034.8 | 830.0 | -160.0 | 0.94 | 680.8 | 275.0 | -428.1 | **0.004** | **0.047** | 0.10 | 0.45 |
| Insulin Concentration (pg/ml) | 787.2 | 831.5 | 10.7 | 1.00 | 1,297.4 | 1,145.8 | -151.6 | 0.68 | 0.54 | 0.08 | 0.49 |
| Insulin Intake (ng/d) | 671.4 | 659.0 | -35.2 | 0.94 | 1,002.9 | 770.7 | -266.7 | 0.21 | 0.34 | 0.23 | 0.47 |
| TNF-α Concentration (pg/ml) | 0.9 | 1.1 | 0.1 | 1.00 | 0.5 | 0.4 | -0.1 | **0.05** | 0.60 | **0.011** | 0.13 |
| TNF-α Intake (pg/d) | 919.6 | 978.7 | -2.0 | 0.81 | 490.0 | 288.4 | -191.4 | 0.08 | 0.46 | 0.11 | 0.46 |
| IL-8 Concentration (pg/ml) | 179.7 | 365.8 | 161.3 | 0.13 | 177.7 | 146.8 | -30.9 | 0.24 | 0.18 | 0.06 | **0.012** |
| IL-8 Intake(ng/d) | 183.7 | 279.7 | 105.4 | 0.08 | 153.5 | 99.5 | -50.4 | 0.12 | 0.73 | 0.24 | **0.010** |
| IL-6 Concentration (pg/ml) | 9.2 | 2.1 | -7.3 | 0.65 | 0.7 | 0.5 | -0.2 | 0.24 | 0.34 | 0.18 | 0.30 |
| IL-6 Intake (ng/d) | 16,036.7 | 1,273.2 | -16,954.0 | 1.00 | 626.4 | 338.7 | -320.7 | 0.21 | 0.28 | 0.23 | 0.23 |
| CRP Concentration (ng/ml) | 114.7 | 101.7 | -5.5 | 0.73 | 156.3 | 130.2 | -7.4 | 0.08 | 0.68 | 0.32 | 0.93 |
| CRP Intake (µg/d) | 112.5 | 119.9 | 17.2 | 0.94 | 111.4 | 98.6 | -24.2 | 0.16 | 0.92 | 0.95 | 0.73 |
| Weight-for-Age z-score | -0.7 | -0.9 | -0.2 | 0.43 | -0.5 | -0.4 | 0.1 | 0.31 | 0.51 | 0.55 | 0.16 |
| Length-for-Age z-score | -1.2 | -1.4 | -0.2 | 0.56 | -0.9 | -0.7 | 0.2 | 0.26 | 0.95 | 0.54 | 0.15 |
| Weight-for-Length z-score | 0.4 | 0.3 | -0.1 | 0.72 | 0.2 | 0.1 | -0.1 | 0.74 | 0.31 | 0.59 | 0.91 |
| Fat Mass Index (kg fat mass/m^2^) | 5.5 | 6.4 | 0.5 | 0.16 | 4.7 | 4.9 | 0.5 | **0.002** | **< 0.001** | 0.11 | 0.87 |
| Fat Free Mass Index (kg fat free mass/m^2^) | 11.5 | 11.8 | 0.2 | 0.94 | 11.1 | 11.0 | -0.1 | 0.34 | 0.96 | 0.49 | 0.42 |
| *Note:* Within each study, values were compared using paired Wilcoxon tests. The two studies were compared using linear mixed-effect models. The bolded values are those that were significantly different (p < 0.05). | | | | | | | | | | | |
